# Fatigue Reliability Analysis of a Compressor Disk Based on Probability Cumulative Damage Criterion

**DOI:** 10.3390/ma13092182

**Published:** 2020-05-09

**Authors:** Jungang Ren, Bingfeng Zhao, Liyang Xie, Zhiyong Hu

**Affiliations:** 1 School of Mechanical Engineering and Automation, Northeastern University, Shenyang 110819, China; renjungang1314@163.com (J.R.); lyxieneu@163.com (L.X.); zhyhu@mail.neu.edu.cn (Z.H.); 2 Key Laboratory of Vibration and Control of Aero-Propulsion System Ministry of Education, Northeastern University, Shenyang 110819, China

**Keywords:** aero engine, variable amplitude load, probability cumulative damage, fatigue reliability, life prediction

## Abstract

The reliability of aero engine has a direct impact on the flight safety of the whole plane. With the continuous improvement of performance requirements of aero engines, the related fatigue and reliability problems also appear. For the fatigue failure characteristics of the typical component (compressor disk) in an aero engine, the fatigue reliability of its multi-site damage structure in service is analyzed by using probability cumulative damage criterion in this paper. The probability distribution definitions of life, damage and damage threshold are discussed and the relationship among them is also introduced by the new proposed criterion. Meanwhile, a method to determine the probability distribution of cumulative damage threshold and probability life prediction is carried out, based on which a hierarchical index system of statistical analysis and reliability modeling principle on the system level is further constructed for compressor disk. At the end of the paper, a certain cruise of fighter plane is analyzed to verify the validity of the new model. Emphasizing the difference between the compressor disk and traditional component, the new reliability analysis model developed in this study is basically reasonable for most of the load histories for the compressor disk, other than the traditional one, especially for the changeable and complex cruise missions.

## 1. Introduction

The reliability of an aero engine has direct effects on the safety of the plane. With the continuous improvement of performance requirements for aero engines, the engine rotors with lighter structure mass, larger workload and higher endurance temperature are required, for which the related fatigue and reliability issues are subsequently exposed [1]. Among various components of aero engine, the related fatigue reliability issue of compressor disk has attracted the attention of scholars due to its complicated structure and the load environment [2,3]. The damage characteristics of multiple parts of the compressor disk can be attributed to its mechanical complexity and stress state. Not only multiple key parts (i.e., the position with high stress or low strength) but also complex mechanical parts on a structural component may be deformed and fractured and then fail in practical applications [4,5,6]. The compressor disk is subjected to the uncertain load and strength, and thus the fatigue life of each damaged part is obviously random. Therefore, to be precise, the life of compressor disk is not equal to the life of weakest part and its damage and probabilistic life prediction is an issue on the system level. In the system with variable amplitude load, there will be statistical correlation between the failure of each key part, and an applicable model should be utilized to predict the system life, as well as its probability distribution [7,8].

In recent years, multi-site damage has caused great concern. Moreover, relevant studies on multi-crack structures have been conducted on different aspects. There are lots of studies reporting on multi-crack structures in different aspects such as residual strength [9], fatigue life [10], fatigue fracture [11], structural integrity [12], etc. Among them, many literatures have studied the fatigue life prediction theory for multi-site damage structures [13,14,15,16], but Xie et al. [17], Wang et al. [18] and Gao et al. [19] pointed out most of the previous studies had been carried out under the deterministic framework, or only simple probability calculation involved. Questions about the fatigue life of a simple part and the probability distribution of fatigue life under a deterministic cyclic load corresponding to a certain reliability are traditionally predicted based on stress-probability life (*P-S-N*) curve [20,21,22,23] or using the stress-strength interference model, for which the influence of load uncertainty on fatigue failure probability needs to be considered [24,25,26]. The influence of load uncertainty on fatigue failure probability can be reflected by the stress-strength interference model. The stress-strength interference model is used to analyze the influence of load uncertainty on fatigue failure probability. For the two kinds of models, the premise of using the latter method is to obtain the probability distribution of fatigue strength corresponding to a certain service-life. Thus, the stress-strength interference model is very difficult to employ widely in engineering applications.

The problem of fatigue failure probability under variable amplitude load history is more complicated, but various studies are limited to specific parts or structures with multi-site damage rarely being taken into account [27,28,29,30]. In the early stage, based on energy criterion, a method to estimate the reliability life of parts under random load was provided by Kliman et al. [31], in which *S-N* curve, cyclic stress-strain curve, standard deviation and probability density distribution of load history, as well as rain-flow counting method are applied to obtain the cyclic load spectrum block. After that, Petryna et al. [32] proposed the probabilistic fatigue damage modeling of reinforced concrete structures and Karadeniz et al. [33] introduced an analysis program for the spectral load uncertainty of offshore engineering structures, respectively. Moreover, Xie et al. [34] further proposed a general method for system reliability analysis under variable amplitude load history based on previous research. According to the failure probability analysis of the system and the probability cumulative damage calculation of the specific damage location, a multi-site damage assessment and fatigue reliability analysis model suitable for aero engine compressor disk in the process of complex tasks is established in this paper.

## 2. Probability Cumulative Damage and Fatigue Life Prediction

In this paper, a method (named median cumulative damage criterion) is introduced to calculate the probability fatigue cumulative damage and probability distribution of critical damage value based on the median *S*–*N* curve, which can be used for the multi-site damage of aero-engine compressor disk. Based on the median cumulative damage criterion, the probabilistic life prediction method of multi-site damage structure is proposed, and the reliability analysis model is established accordingly.

### 2.1. Medians of Expressing Probability Cumulative Damage

The fatigue life at specific cyclic stress level is a random variable, usually obeying the Weibull distribution or lognormal distribution. The distribution of fatigue life under variable amplitude load will show a more complex form. In order to estimate and predict the probability life of components under variable amplitude load, the probability characteristics of fatigue damage and the related cumulation method should be studied. Firstly, the probability distribution of fatigue damage and the expression of its threshold under variable amplitude load was put forward, as well as the calculation method of damage cumulation corresponding to random critical damage.

Under the specified cyclic stress level *s_i_*, the fatigue life of a structure is a random variable with median value ${\bar{N}}_{i}^{}$ and standard deviation $\sigma_{i}$, and the probability density function is expressed as *f_i_*(*N*). After *n_i_* load samples are applied on the structure, the failure probability can be calculated by

(1) $$P\left(N<n_{i}\right)=\int_{0}^{n_{i}}f_{i}\left(N\right)dN$$

In order to facilitate the damage equivalent transformation under different stress levels and the cumulation calculation of probability damage, the probability equivalent transformation between the deterministic stress cycles and random variables of fatigue life should be carried out. By assuming the random variable *N_i_* as a deterministic quantity ${\bar{N}}_{i}^{}$ (equal to the median value of *N_i_*), the stress cycles *n_i_* can be taken as a random variable, represented by the symbol $n_{i}^{r}$, with median value taking the value of the actual cycle number *n_i_*, by which the relationship $P(N<n_{i}^{})=P(n_{i}^{r}>\bar{N})$ can be obtained.

(2) $$\int_{\bar{N}}^{\infty }f_{N,i}\left(n\right)dn=\int_{0}^{n_{i}}f_{n,i}\left(N\right)dN$$

Obviously, since such a transformation can be realized, the probability damage can be calculated based on the median life value and the corresponding random variable of stress cycle. Similarly, according to the equivalence relationship between $n_{i}^{}/N_{i}$~$n_{i}^{r}/{\bar{N}}_{i}$ and $n_{i}^{}/{\bar{N}}_{i}$~$N_{i}^{}/{\bar{N}}_{i}$ in probability calculation, the deterministic cumulative damage threshold “1” can be converted into a random variable of cumulative damage threshold with the deterministic median cumulative damage being employed. Since life *N_i_* is a random variable, the damage *n_i_/N_i_* of stress cycles *n_i_* is also a random variable, expressed as $n_{i}^{r}/{\bar{N}}_{i}$ after the randomization transformation of stress cycles. Thus, the fatigue damage random variable can be calculated based on the life random variable $N_{i}$, as well as by the median life ${\bar{N}}_{i}^{}$.

From the perspective of damage threshold, the failure probability under specified cyclic stress amplitude can be expressed as

(3) $$P\left(n>N\right)=P\left(\frac{n}{\bar{N}}>\frac{N}{\bar{N}}\right)$$

Since $n/\bar{N}$ is defined as median damage and *N* is a random variable, according to Equation (3), the cumulative damage threshold can be interpreted as a random variable. According to the conventional and deterministic linear cumulative damage criterion, the damage threshold is independent of stress level. In order to consider the failure probability, the relationship between threshold of damage random variable and stress level needs to be analyzed firstly in probabilistic meaning. Coincidentally, our previous study [17] has shown that although the stress level has a significant influence on the distribution pattern of life (the general criterion is that low stress and long life correspond to large life dispersion), the dispersion of damage critical value is not as obvious as that of life when the random variable of life with larger dispersion meets the larger life mean under low stress level. It can be roughly considered that the distribution of damage critical value of the same material is independent of the stress level.

Therefore, the probability distribution of cumulative damage threshold can be determined based on the life distribution at any stress level (generally taken as the stress level with the largest number of fatigue test samples).

### 2.2. Probability Cumulative Damage Criterion

The conventional cumulative damage is defined under the condition that the life and load history are both deterministic. Since the life is a random variable, damage caused by the certain load history is also a random variable. To obtain probabilistic life or reliable life, it is necessary to formulate an appropriate probability cumulative damage criterion. As mentioned above, fatigue damage accumulation can be calculated according to the median life value. Then the probability life can be obtained based on the probability distribution of median cumulative damage and cumulative damage threshold, which is the prime target of this section.

Following the linear damage accumulation theory, the median life value under different stress levels is converted to calculate the equal damage stress cycles under corresponding stress levels, which means the fatigue damage generated by *n_j_* cycles under stress *s_j_* can be equivalent to the fatigue damage generated by *n_i_* cycles under stress *s_i_*

(4) $$n_{j}=n_{i}{\bar{N}}_{j}^{}/{\bar{N}}_{i}$$

Based on the assumption that the distribution of damage critical value of the same material is independent of the stress level, the basic property of "same damage leads to the same failure probability" can be further deduced

(5) $$P\left(n_{j}>N_{j}\right)=P\left(n_{i}>N_{i}\right)\Rightarrow P\left(\frac{n_{j}^{}}{{\bar{N}}_{j}}>D_{C}\right)=P\left(\frac{n_{i}^{}}{{\bar{N}}_{i}}>D_{C}\right)$$

Equation (5) is the basis of new probabilistic damage accumulation criterion defined in this study. Based on this basic relationship, the failure probability under variable amplitude cyclic stress is given by

(6) $$p=P\left(\frac{n_{1}}{{\bar{N}}_{1}}+\frac{n_{2}}{{\bar{N}}_{2}}+\cdot \cdot \cdot +\frac{n_{m}}{{\bar{N}}_{m}}>D_{C}\right)=P\left(\sum_{i=1}^{m}\frac{n_{i}}{{\bar{N}}_{i}}>D_{C}\right)$$

This just means that the failure probability under variable amplitude stress is the probability that the linear cumulative damage value calculated based on the median life value is greater than the critical damage random variable.

### 2.3. Probabilistic Fatigue Life Prediction Model

For multi-site damage structures, fatigue damage of each damage site is different under the variable amplitude loads, as well as the damage evolution process, caused by the different stress histories at different damage sites. Supposing that the load history of a structure is composed of *m* stress distributions, the *i*th stress distribution is expressed as *f_i_*(*s*) with *n_i_* load samples. On the basis of the linear cumulative damage calculation formula (Miner rule), the cumulative damage (i.e., the median damage) of component calculated by the median life at each stress level is given by

(7) $$D_{i}=\sum_{i=1}^{m}\left(\int_{s_{0}}^{\infty }f_{i}\left(s\right)\frac{n_{i}}{\bar{N}\left(s\right)}ds\right)$$

where $\bar{N}\left(s\right)$ and *s_0_* are the median *S*-*N* curve equation and fatigue limit of component material, respectively. For the component with *M* damaged sites, the cumulative damage at the *j*th damaged site can be obtained as follows

(8) $$D_{i,j}=\sum_{i=1}^{m}\left(\int_{s_{0}}^{\infty }f_{i,j}\left(s\right)\frac{n_{i,j}}{\bar{N}\left(s\right)}ds\right)$$

where $f_{i,j}\left(s\right)$ and $n_{i,j}$ are the *i*th stress distribution on the *j*th site and the corresponding number of loads, respectively.

By substituting the results into Equation (6), it can be obtained that the failure probability of damaged site *j* is

(9) $$p_{j}=P\left(D_{i,j}>D_{C}\right)=\int_{0}^{D_{i.j}}f\left(d_{C}\right)dd_{C}=F\left(D_{i,j}\right)$$

where *f*(*d_c_*) and *F*(*D_c_*) are respectively the probability density function and cumulative probability distribution function of damage threshold calculated under the selected stress level.

If the life follows two-parameter Weibull distribution at the base stress level, the probability density function can be expressed as

(10) $$f\left(n_{B}\right)=\frac{\beta n_{B}{}^{\beta -1}}{\eta^{\beta }}e^{-{\left(\frac{n_{B}}{\eta }\right)}^{\beta }}$$

where *β* and *η* are shape parameter and scale parameter of Weibull distribution, respectively.

Based on the definition of median damage threshold ($D_{C}=N_{B}^{}/{\bar{N}}_{B}$, ${\bar{N}}_{B}$ is the mean life at the base stress level) and the basic principle of probability distribution for deterministic random variable, the probability density function and cumulative probability distribution function of damage critical value can be obtained as

(11) $$f\left(d_{C}\right)=\frac{{\bar{N}}_{B}\beta {\left({\bar{N}}_{B}d_{C}\right)}^{\beta -1}}{\eta^{\beta }}e^{-{\left(\frac{{\bar{N}}_{B}d_{C}}{\eta }\right)}^{\beta }}$$

(12) $$F\left(d_{C}\right)=1-e^{-{\left(\frac{{\bar{N}}_{b}d_{C}}{\eta }\right)}^{\beta }}$$

Substituting Equations (11) and (12) into Equation (9), in the process of loading, the specific expression form of failure probability of the damaged site *j* can be expressed as

(13) $$p_{j}=\int_{0}^{\sum_{i=1}^{m}\left(\int_{s_{0}}^{\infty }f_{i,j}\left(s\right)\frac{n_{i,j}}{\bar{N}\left(s\right)}ds\right)}\left(\frac{{\bar{N}}_{B}\beta {\left({\bar{N}}_{B}d_{C}\right)}^{\beta -1}}{\eta^{\beta }}e^{-{\left(\frac{{\bar{N}}_{B}d_{C}}{\eta }\right)}^{\beta }}dd_{C}\right)=1-e^{-{\left(\frac{{\bar{N}}_{B}\sum_{i=1}^{m}\left(\int_{s_{0}}^{\infty }f_{i,j}\left(s\right)\frac{n_{i,j}}{\bar{N}\left(s\right)}ds\right)}{\eta }\right)}^{\beta }}$$

According to the probabilistic algorithm of series system, the failure probability and reliability of component with *M* damaged sites are expressed as Equations (14) and (15), respectively.

(14) $$\begin{array}{l} p=1-\prod_{j=1}^{M}\int_{D_{i.j}}^{\infty }f\left(d_{C}\right)dd_{C}=1-\prod_{j=1}^{M}\int_{\sum_{i=1}^{m}\left(\int_{s_{{}_{0}}}^{\infty }f_{i,j}\left(s\right)\frac{n_{i,j}}{\bar{N}\left(s\right)}ds\right)}^{\infty }\left(\frac{{\bar{N}}_{B}\beta {\left({\bar{N}}_{B}d_{C}\right)}^{\beta -1}}{\eta^{\beta }}e^{-{\left(\frac{{\bar{N}}_{B}d_{C}}{\eta }\right)}^{\beta }}dd_{C}\right)\\ =1-\prod_{j=1}^{M}e^{-{\left(\frac{{\bar{N}}_{B}\sum_{i=1}^{m}\left(\int_{s_{{}_{0}}}^{\infty }f_{i,j}\left(s\right)\frac{n_{i,j}}{\bar{N}\left(s\right)}ds\right)}{\eta }\right)}^{\beta }} \end{array}$$

(15) $$\begin{array}{l} R=\prod_{j=1}^{M}\int_{D_{i.j}}^{\infty }f\left(d_{C}\right)dd_{C}=\prod_{j=1}^{M}\int_{\sum_{i=1}^{m}\left(\int_{s_{{}_{0}}}^{\infty }f_{i,j}\left(s\right)\frac{n_{i,j}}{\bar{N}\left(s\right)}ds\right)}^{\infty }\left(\frac{{\bar{N}}_{B}\beta {\left({\bar{N}}_{B}d_{C}\right)}^{\beta -1}}{\eta^{\beta }}e^{-{\left(\frac{{\bar{N}}_{B}d_{C}}{\eta }\right)}^{\beta }}dd_{C}\right)\\ =\prod_{j=1}^{M}e^{-{\left(\frac{{\bar{N}}_{B}\sum_{i=1}^{m}\left(\int_{s_{{}_{0}}}^{\infty }f_{i,j}\left(s\right)\frac{n_{i,j}}{\bar{N}\left(s\right)}ds\right)}{\eta }\right)}^{\beta }} \end{array}$$

## 3. Case Study of a Certain Cruise Mission

**Figure 1:**
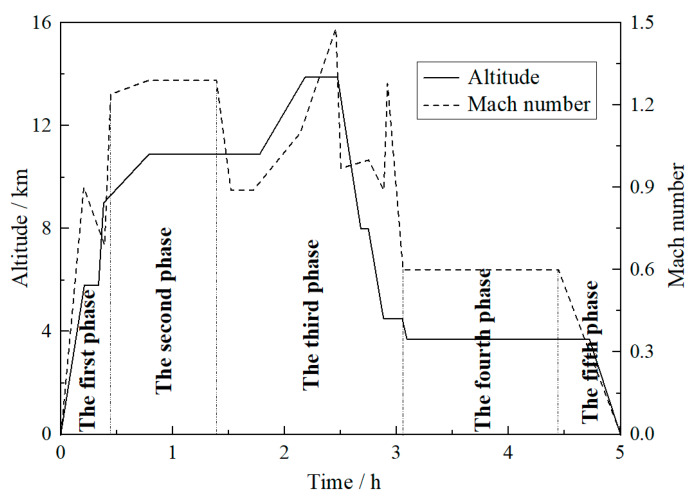
A long cruise curve of the certain type of plane by in-flight refueling.

For the probabilistic fatigue life prediction model proposed in this study, a specific example was employed to verify its applicability in this section. Referring to the published literature [35] and the related mechanics theory, a long cruise curve of the certain type of plane in the Pakistan air force base by in-flight refueling is shown in Figure 1, in which the important parameters, flight altitude and speed of the plane, are described. It is well known that the aero engine can provide power for plane to keep certain altitude and speed. In this process, the output power of aero engine can be obtained indirectly based on the change rates of flight altitude and Mach number. Based on the analysis for change rate of these two parameters in Figure 1, it can be found that the output power of aero engine for the plane is obviously regional during the whole cruise mission. Furthermore, the cruise mission can be roughly divided into five flight cruise phases according to the power state of its engine (work power, namely output power). The specific state of each phase is also shown in Figure 1.

**Figure 2:**
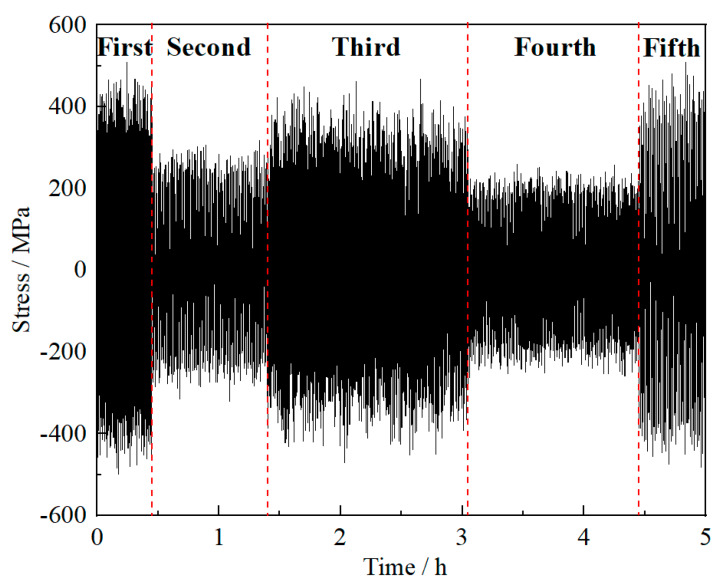
Load spectrum of compressor disk in one cruise mission.

Similarly, based on Figure 1, by observing the cruise characteristics of plane in each phase, the characteristics of aero engine output power in the corresponding phase can be deduced. The first and fifth phases are the take-off and landing phases, respectively. During these phases, the flight speed and altitude of plane changed sharply, but the acceleration of the two indexes was relatively stable. Although the output power of these phases is pretty high, the output remains stable. The second and fourth cruise phases are the conventional high and low altitude cruise phases, respectively. During these phases, the flight speed and altitude of plane remained almost stable. Moreover, the change rates and acceleration of each index in the plane were kept at a low level. The output power of aero engine at these phases was quite small and also stable. The third cruise phase is the increase or decrease flight phase. During this phase, the flight speed and altitude of plane changed frequently, and the acceleration also fluctuated violently. The output power of aero engine at this phase are maintained at a high and fluctuating level. Based on the above analysis, it can be assumed that the load applied on the compressor disk of aero engine in each phase follows a normal distribution, with distribution parameters (mean value *μ*_s_ and standard deviation *σ*_s_) determined by the characteristics of aero engine output power at each cruise phase. The statistical parameters of the spoke hole on the compressor disk (*σ*_s_ is the stress on spoke hole, the same below) in five phases are listed in Table 1. In Table 1, it should be emphasized that the unusual h^−1^ unit is employed due to the hours-long cruise mission, which is investigated in our study. Besides, according to the actual cruise characteristics and service conditions of the aero engine, the rotating speed *v* and working time *t* of the aero engine rotor at the corresponding phase are also given to describe the load number of the compressor disk at each phase, as listed in Table 1. The load spectrum of the compressor disk during a cruise mission can be preliminarily presented by these parameters. As illustrated in Figure 2, there is a significant difference in the load history of the compressor disk at each cruise phase, which requires corresponding segmentation in the calculation process.

**Figure 3:**
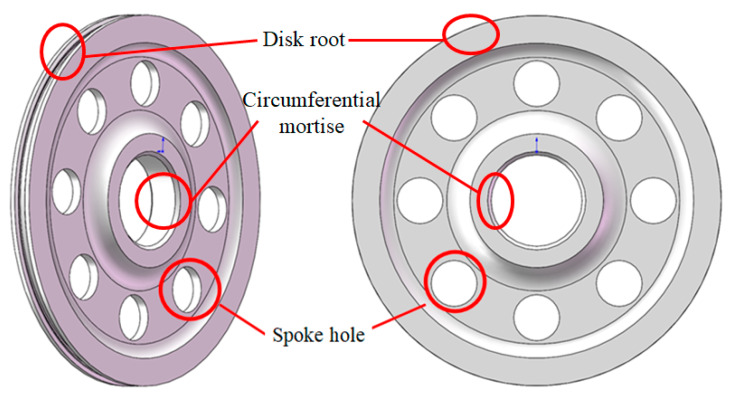
Structural sketch of compressor disk.

**Figure 4:**
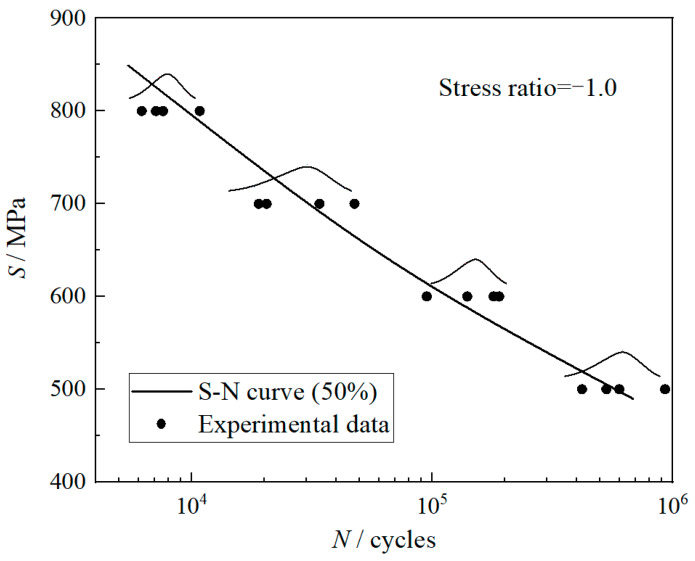
Median *S*–*N* curve of 961 steel obtained by tensile test.

The structural sketch of the compressor disk is shown in Figure 3. According to the analysis, the fatigue failure mainly occurs at the disk root, spoke hole and circumferential mortise. The paper is committed to the fatigue problem research of the spoke hole with the multi-site damages in the eight spoke holes that were considered. In terms of materials, the 961 steel with full complex chemical composition 1Cr11Ni2W2MoV, the most commonly used material of compressor disk, was selected as the object of study. As for the fatigue performance of 961 steel at stress ratio of −1, it has been tested in our previous study [36] and the *S*-*N* curve distribution of 961 steel is shown in Figure 4. Taking the stress level 500 MPa as a reference, it is assumed that the life distribution of material at this stress level is in line with the two-parameter Weibull distribution. The shape parameters and scale parameters of life distribution calculated by the sample clustering theory [37] are 3.4569 and 690230, respectively. In addition, the median value (50% reliability) *S*-*N* curve of material can also be fitted, as shown in Figure 4.

(16) $$s=2095.27\times {\bar{N}}_{}^{-0.106}$$

Then, it can be transformed into the form in Equation (7)

(17) $$\bar{N}\left(s\right)={\left(\frac{s}{2095.27}\right)}^{-9.434}$$

Resulting from the analysis above, the corresponding relationship between the median life of material and stress level (Equation (17)) is substituted into Equation (8) to obtain the cumulative damage on the *j*th spoke hole (damage site) in each cruise phase

(18) $$D_{i,j}\left(t\right)=\{\begin{array}{c} D_{1,j}\left(t\right)=\int_{s_{0}}^{\infty }f_{1,j}\left(s\right)\frac{t\times v_{1}}{{\left(\frac{s}{2095.27}\right)}^{-9.434}}ds,\;\mathrm{Frist}\;\mathrm{phase}\\ D_{2,j}\left(t\right)=\int_{s_{0}}^{\infty }f_{2,j}\left(s\right)\frac{\left(t-4.5\right)\times v_{2}}{{\left(\frac{s}{2095.27}\right)}^{-9.434}}ds,\;\mathrm{Sec}\mathrm{ond}\;\mathrm{phase}\\ D_{3,j}\left(t\right)=\int_{s_{0}}^{\infty }f_{3,j}\left(s\right)\frac{\left(t-1.4\right)\times v_{3}}{{\left(\frac{s}{2095.27}\right)}^{-9.434}}ds,\;\mathrm{Third}\;\mathrm{phase}\\ D_{4,j}\left(t\right)=\int_{s_{0}}^{\infty }f_{4,j}\left(s\right)\frac{\left(t-3.05\right)\times v_{4}}{{\left(\frac{s}{2095.27}\right)}^{-9.434}}ds,\;\mathrm{Fourth}\;\mathrm{phase}\\ D_{5,j}\left(t\right)=\int_{s_{0}}^{\infty }f_{5,j}\left(s\right)\frac{\left(t-4.45\right)\times v_{5}}{{\left(\frac{s}{2095.27}\right)}^{-9.434}}ds,\;\mathrm{Fifth}\;\mathrm{phase} \end{array}$$

where *v_i_* is the rotating speed in the *i*th cruise phase, and the value is shown in Table 1. It can be obtained from Equation (18) that the cumulative total damage on the *j*-spoke hole (damage site) in a cruise mission (including five cruise phases) is given by

(19) $$D_{j}=\sum_{i=1}^{5}D_{i,j}\left(t_{i}\right)=\sum_{i=1}^{5}\int_{s_{0}}^{\infty }f_{i,j}\left(s\right)\frac{t_{i}\times v_{i}}{{\left(\frac{s}{2095.27}\right)}^{-9.434}}ds$$

where *t_i_* is the cruise time of the *i*th cruise phase, and the specific value is shown in Table 1. For easy figures, this study adopts the "6*σ* criterion" to make a finite amendment to the upper limit of the integral in Equation (19). The modified result is shown as

(20) $$D_{j}=\sum_{i=1}^{5}D_{i,j}\left(t_{i}\right)=\sum_{i=1}^{5}\int_{s_{0}}^{\mu_{si}+6\sigma_{si}}f_{i,j}\left(s\right)\frac{t_{i}\times v_{i}}{{\left(\frac{s}{2095.27}\right)}^{-9.434}}ds$$

where *μ*_si_ and *σ*_si_ are the mean and standard deviation of load distribution in the *i*th cruise phase, respectively. By substituting the analysis results into Equations (14) and (15), the failure probabilities and reliabilities of single spoke hole *j* and compressor disk with 8 spoke holes in a cruise mission can be expressed by

(21) $$\{\begin{array}{c} p_{j}=1-e^{-{\left(\frac{{\bar{N}}_{B}\times \sum_{i=1}^{5}\int_{s_{0}}^{\mu_{si}+6\sigma_{si}}f_{i,j}\left(s\right)\frac{t_{i}\times v_{i}}{{\left(\frac{s}{2095.27}\right)}^{-9.434}}ds}{\eta }\right)}^{\beta }}\\ R_{j}=e^{-{\left(\frac{{\bar{N}}_{B}\times \sum_{i=1}^{5}\int_{s_{0}}^{\mu_{si}+6\sigma_{si}}f_{i,j}\left(s\right)\frac{t_{i}\times v_{i}}{{\left(\frac{s}{2095.27}\right)}^{-9.434}}ds}{\eta }\right)}^{\beta }} \end{array}$$

(22) $$\{\begin{array}{c} p=1-\prod_{j=1}^{8}e^{-{\left(\frac{{\bar{N}}_{B}\times \sum_{i=1}^{5}\int_{s_{0}}^{\mu_{si}+6\sigma_{si}}f_{i,j}\left(s\right)\frac{t_{i}\times v_{i}}{{\left(\frac{s}{2095.27}\right)}^{-9.434}}ds}{\eta }\right)}^{\beta }}\\ R=\prod_{j=1}^{8}e^{-{\left(\frac{{\bar{N}}_{B}\times \sum_{i=1}^{5}\int_{s_{0}}^{\mu_{si}+6\sigma_{si}}f_{i,j}\left(s\right)\frac{t_{i}\times v_{i}}{{\left(\frac{s}{2095.27}\right)}^{-9.434}}ds}{\eta }\right)}^{\beta }} \end{array}$$

**Figure 5:**
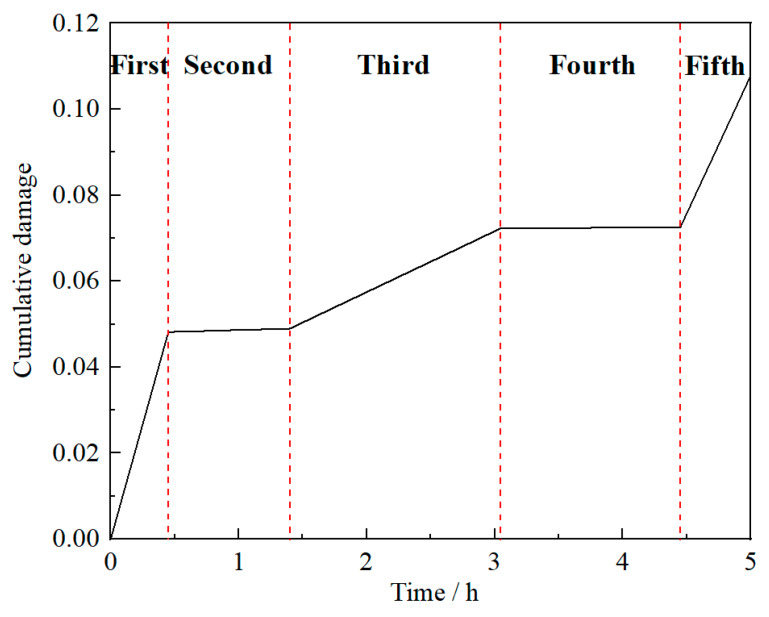
Relationship between cumulative damage and cruise time.

**Figure 6:**
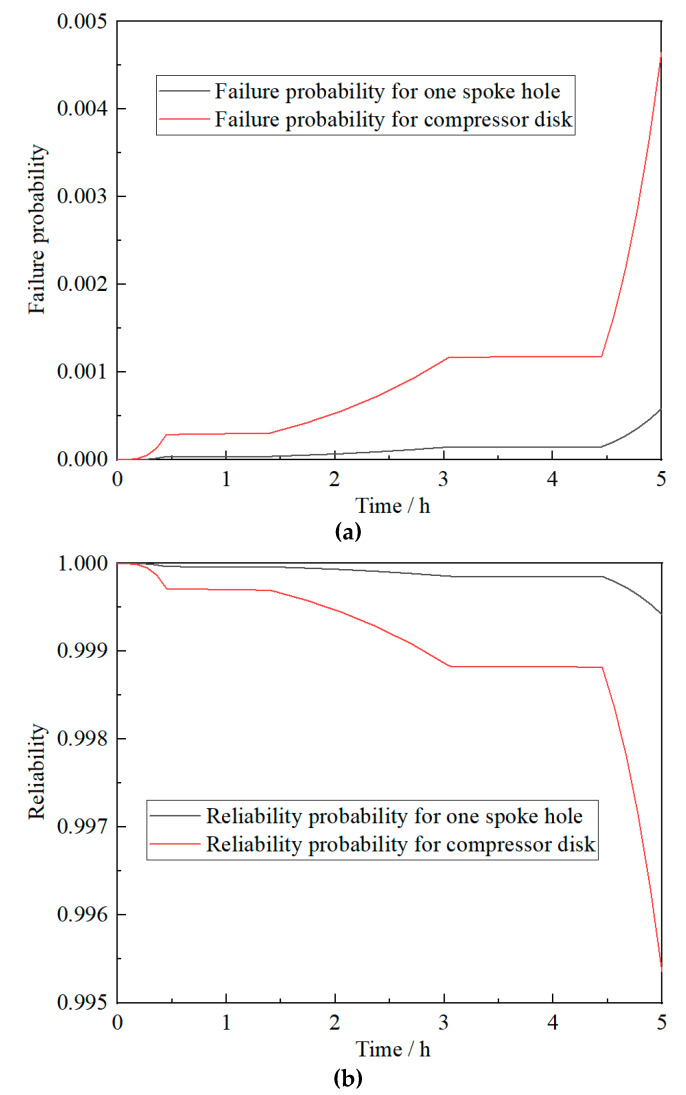
Failure probability (**a**) and reliability (**b**) curves for one spoke hole or the compressor disk in one cruise mission.

Based on the previous test data analysis (in Figure 4) and Equation (17), the following results can be calculated: *β* = 3.4569, *η* = 690230 and ${\bar{N}}_{B}$ = 742156. Firstly, according to Equation (18), the cumulative damage changes at a single spoke hole in a cruise mission can be obtained, as shown in Figure 5. From Equations (21) and (22), the failure probabilities and reliabilities of a single spoke hole and the whole compressor disk in a cruise mission can be further derived. The results are shown in Figure 6.

According to the calculation results, the fatigue life of the compressor disk is much lower than that of a spoke hole (damaged site) under the given failure probability due to the effect of uncertainty life. Apparently, there is an obvious difference between the lives of structure and one damaged site when the randomness becomes larger and service life becomes longer.

## 4. Discussion

**Figure 7:**
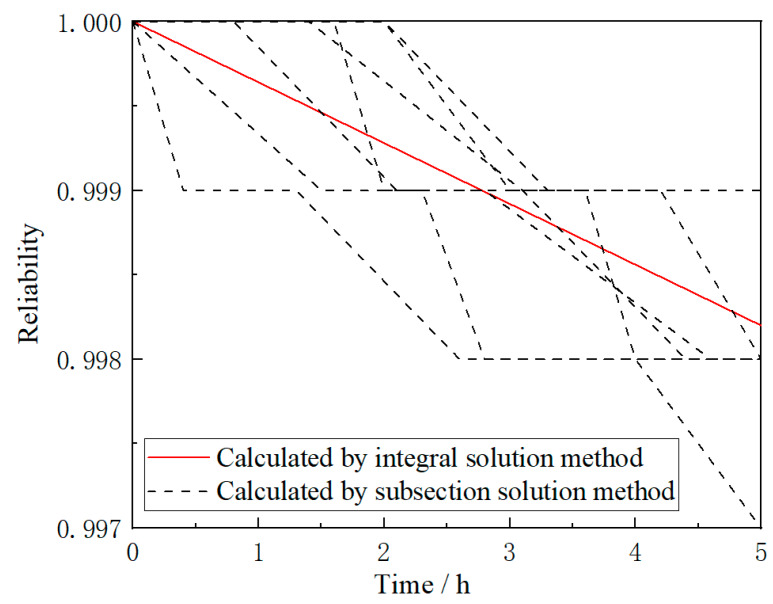
Reliabilities calculated by integral solution method and subsection solution method in different cruise missions.

Moreover, it can be concluded that the reliability of a compressor disk calculated by an integral solution method remains constant as long as the statistical average parameters of all phases in these cruise missions are the same. However, reliabilities calculated by the subsection solution method for these cruise missions are different as the results are obtained based on the parameters of all independent phases in these cruise missions. Seen from Figure 7, reliabilities calculated by the subsection solution method and integral solution method for some different cruise missions with the same statistical average parameter are listed. Also, in Figure 7, it can be seen that only one result can be calculated by an integral solution method for these cruise missions, but different results can be obtained by subsection solution method according to different combinations of cruise missions. Based on further analysis and comparison, for one compressor disk with the same statistical average parameters of all phases in one cruise mission, there are infinitely many combinations of flight phases corresponding to it, and reliabilities of the compressor disk with different flight phase combinations will be clearly different, which indicates that the subsection solution method is more reasonable for reliability analysis of compressor rotor blade system with different flight phases.

**Figure 8:**
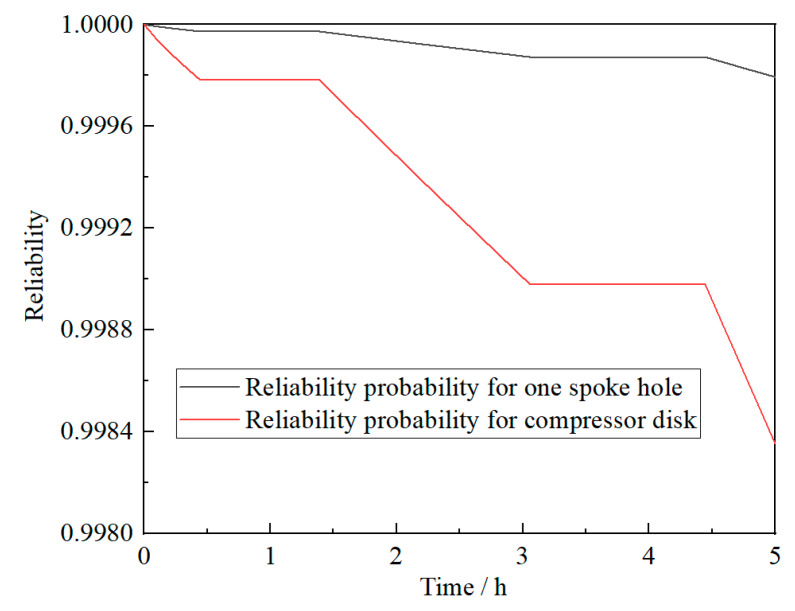
Reliability calculated by the stress-strength interference model for one spoke hole or the compressor disk in one cruise mission.

**Figure 9:**
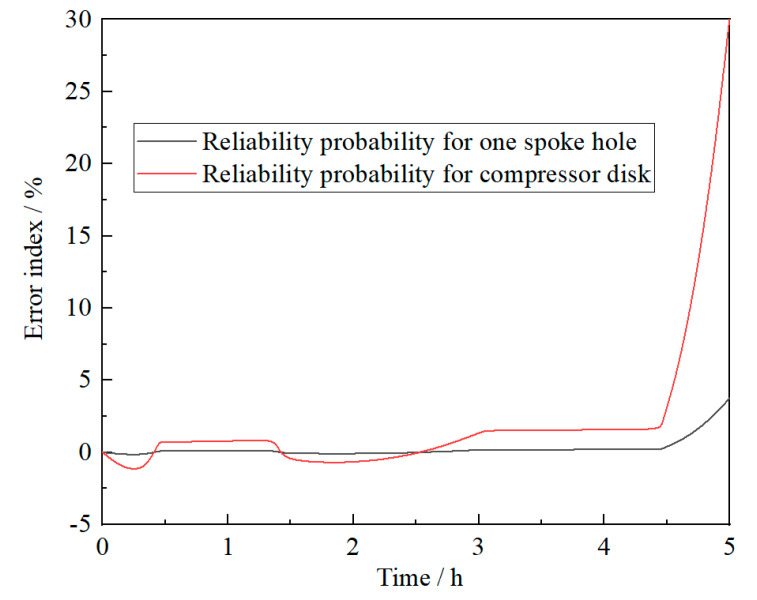
Error index of the stress-strength interference model compared with the new model in one cruise mission.

In order to fully embody the difference of the subsection solution method proposed in this paper, the analysis result of the stress-strength interference model is also calculated to compare with the result of the new model. In the calculation process of the stress-strength interference model, assume that the degeneration of material strength follows an exponential criterion, which can be obtained from Figure 4. Reliabilities of the same compressor disk and spoke hole in one cruise mission, calculated by stress-strength interference model, are described in Figure 8. According to the theoretical analysis, it can be known that the new reliability analysis model is more accordant with the practical cruise characteristics of compressor disk. The result of the proposed model is more reasonable and can be taken as the benchmark for precision comparison accordingly. In order to quantify the deviation of the stress-strength interference model concretely, the error index *E* is defined with the results of the new model reference.

(23) $$E=\frac{R_{1}-R_{0}}{R_{0}\left(1-R_{\min}\right)}\times 100\%$$

where *R_0_* is the reliability of the new method; *R_1_* is the reliability of the stress-strength interference model; *R_min_* is the reliability limit of compressor disk in the design period, *R_min_* = 0.99 here. The error index of the stress-strength interference model, compared with the new method, in one cruise mission is shown in Figure 9. From Figure 9, it can be seen that the stress-strength interference model underestimates the actual damage of compressor disk, which means the component will prematurely fail compared with the predicted life. It can also be found from Figure 9 that the error index of the stress-strength interference model increases with the increase of cruise time.

## 5. Conclusions

To analyze the reliability of compressor disk before the incidents occur, a new reliability analysis model is developed in the paper. The major characteristics of this study are as follows:

1. A method to calculate the probability fatigue cumulative damage and probability distribution of critical damage value based on the median *S*–*N* curve is introduced, based on which the probabilistic life prediction method of multi-site damage structure is proposed, and the reliability analysis model is established accordingly.
2. Due to the complex working environment applied to aero engine, a subsection solution method is innovatively proposed by dividing a cruise mission into several phases with different loading environments.

In the using process, only median *S–N* curve and life distribution under a certain stress level are required for material performance data, for which it is convenient for engineering application. At the end of the paper, a certain cruise of fighter plane is analyzed to verify the validity of the new model. Emphasizing the difference between compressor disk and traditional component, the new reliability analysis model developed in this study is basically reasonable to most of the load history for a compressor disk than the stress-strength interference model, especially for the changeable and complex cruise mission. Moreover, further studies also show that the proposed model can provide a more secure result for the compressor disk in long cruise missions.

## Figures and Tables

**Table 1:** Statistical parameters of the compressor disk in five phases.

Parameters	First Phase	Second Phase	Third Phase	Fourth Phase	Fifth Phase
*μ*_s_/MPa	400	250	325	200	400
*σ*_s_/MPa	40	30	55	25	38
*v*/10^5^ × rh^−1^	9.0	6.0	8.4	7.2	5.4
*t*/h	0.45	0.95	1.65	1.40	0.55
